# Chemical Constituents of Stems and Leaves of *Tagetespatula* L. and Its Fingerprint

**DOI:** 10.3390/molecules24213911

**Published:** 2019-10-30

**Authors:** Yu-Meng Wang, Xiao-Ku Ran, Muhammad Riaz, Miao Yu, Qian Cai, De-Qiang Dou, Ahmed M. Metwaly, Ting-Guo Kang, De-Cheng Cai

**Affiliations:** 1 College of Pharmacy, Liaoning University of Traditional Chinese Medicine, Dalian 116600, China; 2 Department of Pharmacy, Shaheed Benazir Bhutto Sheringal Dir Upper, Khyber PakhtoonKhwa 18000, Pakistan; 3 Department of Pharmacognosy, Faculty of Pharmacy, AI-Azhar University, Cairo 11884, Egypt; 4 Dalian Wuzhou Holy Herb Scientific and Techonological Co. Ltd., Dalian 116600, China

**Keywords:** *Tagetespatula* L., benzofurans, flavonoids, fingerprint

## Abstract

*Tagetespatula* L. is a widely cultivated herbal medicinal plant in China and other countries. In this study, two new 2, 3-dihydrobenzofuran glucosides (**1**, **2**) and fourteen known metabolites (**3**–**16**) were isolated from the stems and leaves of *T. patula* (SLT). The chemical structures of the isolated compounds were characterized comprehensively based on one- and two-dimensional NMR spectroscopy and high resolution mass spectrometry. Absolute configurations of compounds **1** and **2** were determined by ECD calculations. Compounds **1** and **2** exhibited moderate in vitro inhibitory activities against human gastric cancer cell lines (AGS) with IC_50_ values of 41.20 μmol/L and 30.43 μmol/L, respectively. The fingerprint profiles of stems and leaves of *T. patula* with three color types of flowers (Janie Yellow Bright, Jinmen Orange, Shouyao Red and Yellow color) were established by high-performance liquid chromatography (HPLC). Ten different batches of stems and leaves were examined as follow: Shouyao Red and Yellow color (1, 2, 3), Janie Yellow Bright (4, 5, 6, 7) and Jinmen Orange (8, 9, 10). Twenty-two common peaks were identified with similarity values ranging from 0.910 to 0.977. Meanwhile, the average peak area of SLT in the three types of flowers was different and it was the highest in Janie Yellow Bright.

## 1. Introduction

Tagetes genus belongs to the family Compositae (Asteraceae) and comprises about 55 different species including *Tagetespatula* Linn. (French marigold) which is an annual well-known ornamental and medicinal plant distributed worldwide [1]. *T. patula*, which first originated in Mexico, exhibits cold and drought-resistant ability and, generally, grows on mountains at an altitude of 1350 m [2].

**Figure 1:**
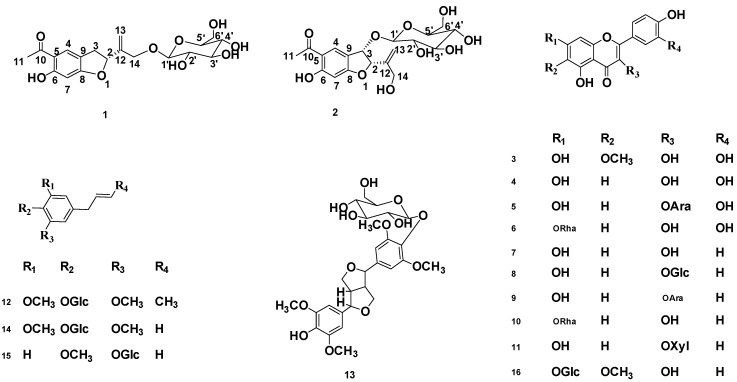
Structure of the isolated compounds from *T. patula*.

**Figure A1:**
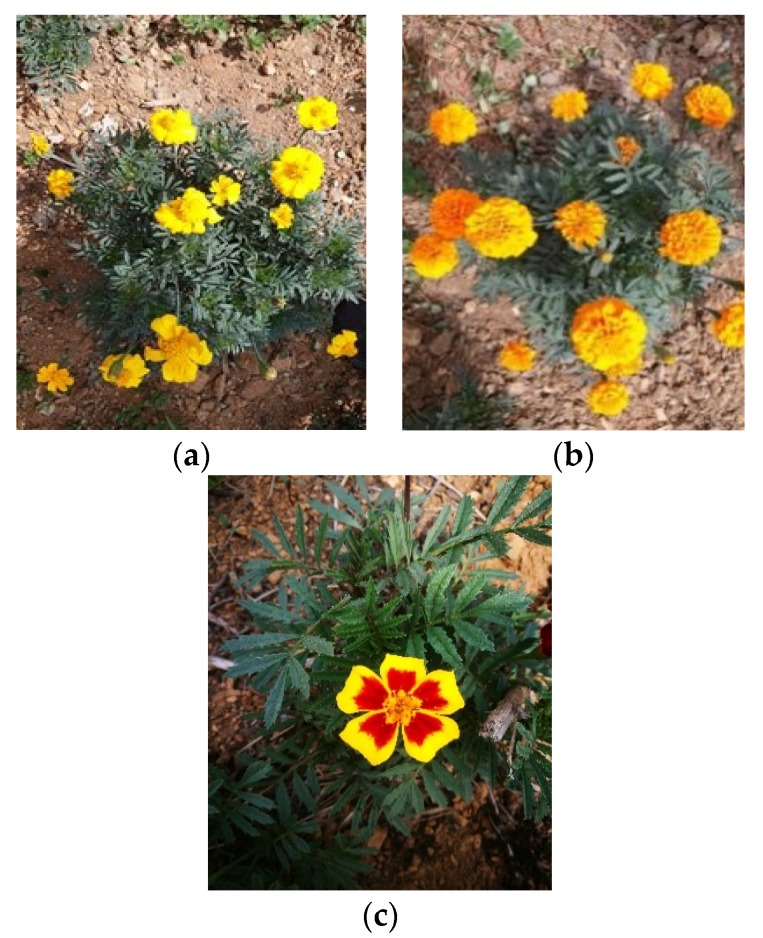
(**a**) Janie Yellow Bright, (**b**) Jinmen Orange, (**c**) Shouyao Red and Yellow color.

Currently, in China, *T. patula* is a common ornamental and medicinal plant and has been cultivated throughout the country. Traditionally, *T. patula* was used to treat various diseases, such as cough, colic, constipation, diarrhea, rheumatism, and eye problems. Today, the plant is specially used as an antimicrobial, antiseptic, blood purifying, and diuretic agent [3,4]. The flowers of *T. patula* are edible and used in refreshing drinks [5]. Previous phytochemical investigations of flowers of *T. patula* were mainly focused on the flavonoids [6,7], steroids [8], and thiophenes [9]. Its roots and rhizomes contained higher contents of thiophenes [10] and benzofurans [11]. In this study, we report the isolation and structural elucidation of two new euparin glycosides and 14 known compounds (Figure 1) using different chromatographic techniques. The chemical structures of the isolated compounds have been determined with different spectroscopic techniques including one-dimensional (1D) and two-dimensional (2D) NMR experiments, HR-ESI-MS analysis, as well as ECD. Furthermore, we established an effective fingerprint method for comparison and quality evaluation of SLT with three color types of flowers (Figure A1).

## 2. Results and Discussion

### 2.1. Structural Elucidation of the Isolated Compounds

**Figure 2:**
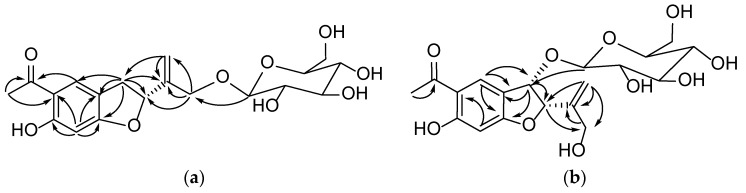
HMBC correlations of compounds **1** (**a**) and **2** (**b**).

**Figure 3:**
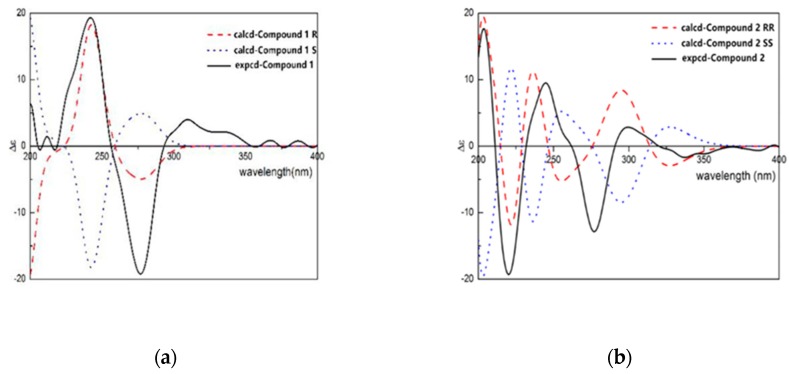
Caculated and experimental ECD spectra of compound **1** (**a**) and **2** (**b**).

Compound **1** was obtained from the 95% EtOH extract of the *T. patula* L as a white amorphous powder. The molecular formula was determined as C_19_H_24_O_9_ byHR-ESI-MS showing a pseudomolecular ion peak at m/z 397.1399 [M+H]^+^ correspondent to of. The ^1^H-NMR spectrum of compound **1** showed resonances of two dd peaks at δ_H_ 3.38 (dd, *J* = 8.0 and 15.5 Hz, H-3) and 3.12 (dd, *J* = 8.0 and 15.5 Hz, H-3) coupled with a triplet signal at δ_H_ 5.48 (t, *J* = 8.5 Hz, H-2), which are the characteristic signals of 2, 3-dihydrobenzofuran derivatives [12]. In addition, one aromatic acetyl methyl group with chemical shift of δ_H_ 2.54 ppm (3H, s, H-11), two singlet aromatic protons at δ_H_ 7.68 (s, H-4) and 6.27 (s, H-7) were also observed. Two downfielded signals at δ_H_ 5.28 and 5.32 assignable to vinylic protons (2H, s, H-13) and two doublets at δ_H_ 4.22 and 4.49 (d, *J* = 10.0 and 12.8 Hz, H-14) were also observed. The ^13^C-NMR spectrum of compound **1** showed signals for nineteen carbons, of which eight were speculated to be from the benzofuran skeleton. The assignment of ^13^C-NMR signals of compound **1** was assisted by the analysis of the HSQC spectrum. The carbon signal at δ_C_ 204.2 (C-10) and δ_C_ 26.4 (C-11) indicated the presence of an acetyl moiety, while the signals at δ_C_ 145.8 and δ_C_ 113.9 could confirm the presence of a double bond. Analysis of the ^13^C-NMR data (Table 1) clearly indicated that compound **1** was closely related to the known compound 2, 3-dihydro-14-isobutyryloxyeuparin and the major difference was that C-14 in compound **1** (δ_C_ 70.0) was shifted downfield as compared with that known compound (δC 62.3) [13]. In addition, the HMBC correlation between the signals at δ_H_ 4.28 (1H, d, *J* = 4.3Hz, glu-1) and δ_C_ 70.0 (C-14) suggested that the attachment of a sugar moiety at C-14 of aglycone (Figure 2). To determine the absolute configuration of compound **1**, the ECD method was used to determine the configuration of C-2 of compound **1**. ECD was calculated by the time-dependent density functional theory (TD-DFT) method at the B3LYP/6-31G+(d, p) level utilizing the conductor polarizable continuum model (CPCM) in methanol. By comparing the calculated ECD spectral data with that of experimental data of compound **1**, the absolute configuration at C-2 was assigned to be R (Figure 3). Thus, the structure of comppound **1** was characterized as 1-{(2R)-2-[1-(*β*-D-glucopyranoside) vinyl]-6-hydroxy-1-benzofuran-5-yl}-ethanone. It is a new compound and named as 2H*β*, 3-dihydro-euparin-14-O-*β*-D-glucoside.

Compound **2** was isolated as a yellow crystalline amorphous powder and its molecular formula C_19_H_24_O_10_ was determined by HR-ESI-MS showing a pseudo molecular ion peak at m/z 435.1269 [M+Na]^+^. The ^1^H-NMR spectrum of compound **2** displayed the presence of the signals of two singlet aromatic protons at δ_H_ 8.18 (1H, s, H-4) and 6.30 (s, H-7), an AB type of methylenic signals at δ_H_ 4.23, 4.16 (2H, d, *J* = 10.0 Hz and *J* = 15.0 Hz, H-14), one singlet acetyl group at δ_H_ 2.61 (3H, s, H-11), two coupled heterocyclic protons at δ_H_ 5.33 (1H, d, *J* = 5.0 Hz, H-2) and 5.17 (1H, d, *J* = 5.0 Hz, H-3), two germinal olefinic protons at δ_H_ 5.41 and 5.36 (2H, each s, H-13), and one anomeric proton at δ_H_ 4.54 (1H, d, *J* = 5.0 Hz, H-1′). The ^13^C-NMR spectrum of compound **2** showed nineteen carbon signals, of which eight from benzofuran skeleton, and the determination was assisted by analysis of the HSQC spectrum. Signals at δ_C_ 205.0 (C-10) were assigned to a carbonyl carbon. Comparison of the ^1^H and ^13^C-NMR spectrum data of compound **2** (Table 1) with known compounds 1-{(2R*,3S*)-3-(*β*-D-glucopyranosyloxy)-2,3-dihydro-2-[1-(hydroxymethyl)vinyl]-1-benzofuran-5-yl}-ethanone showed their close structural relationship [14]. Differences were observed for the C-6 which resonated as an oxygenated carbon in the ^13^C-NMR spectrum. In addition to the two singlet signals of H-4 and H-7, the ^1^H-NMR spectrum were found. A *β*-glucopyranosyl was elucidated to be attached to C-14 based on HMBC correlations of H-1′ with C-13 (Table A1). The H-2 and H-3 were deduced to be in a cis conformation via NOESY correlations. The ECD data were calculated based on time-dependent density functional theory (TDDFT) (Figure 3). The absolute configurations of C-2 and C-3 were determined as (2R, 3R) by comparison of the calculated ECD spectra (Figure 3). Thus compound **2** was assigned as 1-{(2R,3R)-3-(*β*-D-glucopyranoside)-2,3-dihydro-2-[1-(hydroxymethyl)vinyl]-6-hydroxy-1-benzofuran-5-yl}-ethanone. It is also a new compound and named as 14-hydroxy-2,3-dihydro -euparin-3-O-*β*- D-glucoside.

Another fourteen known compounds were identified as patuletin (**3**) [15], quercetin (**4**) [16], quercetin-3-O-α-L-arabinopyranoside (**5**) [17], quercetin-7-O-α-L-rahmnoside (**6**) [18], kaempferol (**7**) [19], kaempferol-3-O-*β*-D-glucoside (**8**) [20], kaempferol-3-O-α-L-arabinopyranoside (**9**) [21], kaempferol-7-O-α-L-rahmnoside (**10**) [22], kaempferol-3-O-*β*-D-xylopyranoside (**11**) [23,24], 1-*β*-D-glucopyranosyl-2, 6-dimethoxy-4-propenylphenol (**12**) [25], syringaresinol-4′-O-*β*-D-glucoside (**13**) [26], 4-allyl-2, 6-dimethoxy phenyl glucoside (**14**) [27], 2-methoxy-4-(2-propenyl) phenyl-*β*-D-glucoside (**15**) [28], and 6-methoxy–kaempferol-7-*β*-D-glucoside (**16**) [29], based on the analysis of their 1D and 2D NMR, MS, as well as other spectroscopies. 

### 2.2. HPLC Fingerprints and Similarity Analysis of the Various SLT Samples

**Figure 4:**
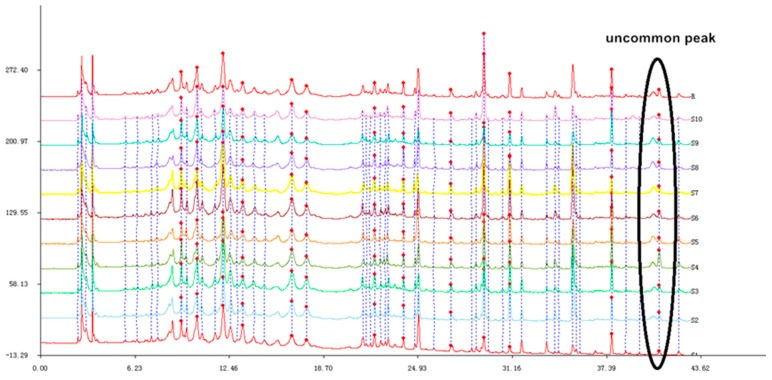
The chromatographic fingerprint of 10 *T. patula* samples (S1–S10).

**Figure 5:**
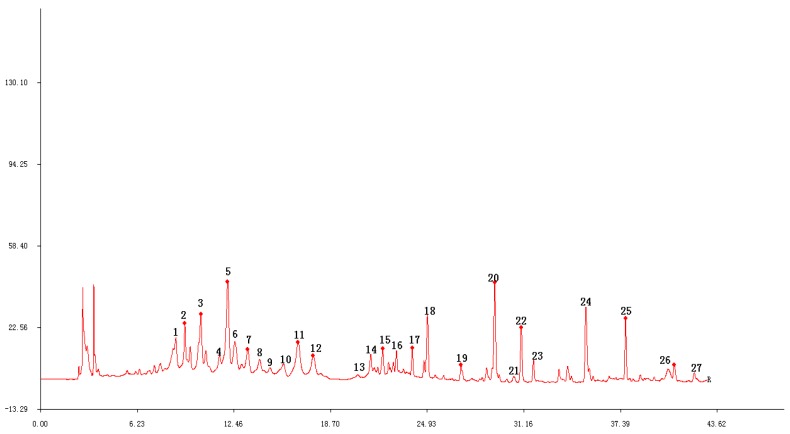
Compound **12**: Quercetin-3-O-α-L, arabinopyranoside; **13**: kaempferol-3-O-*β*-D, glucoside; **14**: kaempferol-3-O-α-L, arabinopyranoside; **15**: kaempferol-3-O-*β*-D, xylopyranoside; **16**: quercetin-7-O-α-L, rahmnoside; **17**: kaempferol-7-O-α-L, rahmnoside; **18**: patuletin; **19**: kaempferol.

The following ten different batches of stems and leaves of *T. patula* (SLT) were examined: Shouyao Red and Yellow color (1, 2, 3), Janie Yellow Bright (4, 5, 6, 7), and Jinmen Orange (8, 9, 10). The chromatographic fingerprint of (SLT) (Figure 4) exhibited 22 distinct peaks from the common patterns. These chromatograms were very similar apart from the differences in peak areas and lackingof common peak 26. Among these chromatograms, patuletin was chosen as the reference peak to calculate the RRT, because it has moderate retention time, a clear shape, and a high peak area. Eight common peaks (peak 12, 13, 14, 15, 16, 17, 18, and 19) were chosen as the “characteristic fingerprint peaks” to represent the characteristics of the SLT and identified as quercetin-3-O-α-L, arabinopyranoside; kaempferol-3-O-*β*-D, glucoside; kaempferol-3-O-α-L, arabinopyranoside; kaempferol-3-O-*β*-D, xylopyranoside; quercetin-7-O-α-L, rahmnoside; kaempferol-7-O-α-L, rahmnoside; patuletin; and kaempferol, respectively. The compounds were identified by comparing their retention time with the reference substances (Figure 5). The new compounds were not recognized due to low peak area. After analyzing, the result of similarity were evaluated for all the samples using to the Similarity Evaluation System for Chromatographic Fingerprint of Traditional Chinese Medicine (Version 2004A). The RPA of characteristic peaks relative to the reference peak were calculated and are shown in Table 2. The RPA data of the characteristic peaks were used to reflect the quantitative expression of the present HPLC fingerprint of the *T. patula*. The average peak area of patuletinin Janie Yellow Bright was 222.7, followed by the Jinmen Orange type (218.4) and ShouyaoRed and Yellow color (212.8). As shown in Table 3, the comparison between the original chromatograms and the average chromatograms of all samples presented similarity values ranging from 0.910 to 0.979, denoting that the quality of all samples was reliable and stable, however, there is a slight difference in various types of samples. Different types of *T*. *patula* should be distinguished by variant or ecological type. The results of the precision, stability, and repeatability rates are shown as supplementary data in (Table A2) and the picture of the three types of flower of SLT are shown in (Figure A1).

### 2.3. Cytotoxicity Assay

The isolated compounds from *T. patula*were evaluated for their cytotoxicity against human gastric cancer cell (AGS). The cytotoxicity assay was performed using the MTT assay method. Human gastric cancer cell (AGS) were obtained from the Cell Bank of Type Culture Collection of the Chinese Academy of Sciences, Shanghai Institute of Cell biology. Compounds **1** and **2** displayed moderate cytotoxicity against human gastric cancer cell (AGS) with IC_50_ values of 41.20 μmol/L and 30.43 μmol/L, respectively, (Table 4). 5-Fluorouracil was used as a positive control and showed an IC_50_ value of 11.79 μmol/L.

## 3. Materials and Methods

### 3.1. General Experimental Procedures 

Optical rotations were measured with a Perkin-Elmer Model 241 polarmeter (Perkin Elmer, Inc. Waltham, MA, USA). The high-resolution electrospray ionization mass spectroscopy (HR-ESI-MS) as was acquired using a Waters Xevo Q-TOF (Waters Corporation, Milford, MA, USA). ECD spectra were recorded on a BioLogic MOS-450 spectrometer. Semipreparative HPLC was performed using a HITACHI 7100 (Hitachi Corporation, Tokyo, Japan) equipped with a YMC-Pack ODS-A, 10 × 250 mm column (YMC, Kyoto, Japan) and detections were performed with a VWD detector at 210 nm. Column chromatography was performed using silica gel (200−300 mesh, Marine Chemical Factory, Qingdao, China). Prepared silica gel G plates (Marine Chemical Factory, Qingdao, China) were used for TLC analysis. Acetonitrile (HPLC grade) was purchased from Duksan (Anshan, Kyonggi, Korea). 

### 3.2. Plant Material

The aerial part of *T. patula* was collected in Dalian, Liaoning province, China, on March 2016 and authenticated by Professor Bing Wang, the Liaoning University of Traditional Chinese Medicine, China.

### 3.3. Extraction and Isolation

The air-dried stems and leaves (15.0 Kg) of T. patula was refluxed with 60% EtOH for three times (3 × 120 L, 2 h each) at low temperature (45 °C), then filtered and the solvent was evaporated in vacuum to furnish the crude extract. Then, it was partitioned with petroleum and ethyl acetate successively several times to give the ethyl acetate layer and H_2_O layer which was subjected to column chromatography (CC) over AB-8 macroporous adsorptive resins and eluted with EtOH/H_2_O (0:100, 60:40 and to 95:5). The 60% EtOH eluate and ethyl acetate layer (total 300 g) were combined to column chromatography on silica gel and eluted with CH_2_CL_2_/MeOH (100:0 to 0:100) to afford six fractions (Fr. A1-A6). Fr. A4 was passed over a silica gel column and eluted with CH_2_CL_2_/MeOH (100:0 to 0:100) to give six fractions (Fr. B1-B6). Compound **3** (27.9 mg) was further crystallized in MeOH from Fr. B3 (CH_2_CL_2_/MeOH, 20:1). Then, Fr. B4 (eluted with CH_2_CL_2_/MeOH, 10:1) was subjected to an ODS column and eluted with MeOH/H_2_O. The 30% fraction from ODS column was 927 mg and gave six fractions C1-6. Fraction C1 was further purified by HPLC (eluted with MeOH/H_2_O, 45:55) to give compound **2** (8 mg) and compound **16** (9 mg). Similarly, Fraction C3 was further subjected to an ODS column and gave three subfractions D1-D3. Fraction D1 was purified on an HPLC (eluted with MeOH/H_2_O, 37:63) to afford compound **12** (8 mg). In the same way, compound **13** (14.8 mg) was isolated from fraction D2 by HPLC (eluted with MeOH/H_2_O, 40:60), whereas compound **1** (8.2 mg), compound **14**, and compound **15** (8 mg) were isolated from fraction D3 by an HPLC (eluted with MeOH/H_2_O, 42:58). Finally, compound **4** (9 mg) was obtained from Fraction B6. Fr. A6 was applied to a silica gel (CH_2_CL_2_/MeOH from 15:1 to 0:100) to give seven subfractions E1-E7. Fraction E2 was further passed through polyamide column (CH_2_CL_2_/MeOH from 1:0 to 0:1) to obtain eight fractions F1-F8. Fraction F4 was further purified by ODS column equipped with HPLC eluting with MeOH/H_2_O (45:55), resulting in optically pure compounds of **5** (16 mg), **6** (18 mg), **8** (34 mg), **11** (10 mg). In the same manner, **7** (12 mg), **9** (10 mg), **10** (20 mg) were isolated from fraction F5. 

### 3.4. Acid Hydrolysis of Compounds **1** and **2**

Compounds **1** and **2** (2 mg each) were hydrolyzed with 10% HCL-dioxane (1:1, 1 mL) for 4 h at 80 °C in a water bath. The mixture was neutralized with Ag2CO3 and extracted with CH2CL2. After the aqueous layer was condensed, both of them were examined by HPLC and compared with authentic samples. Analytical HPLC was performed on Agilent ZORBAX NH2 column (5 μm, 4.6 mm × 150 mm) with isocratic elution using MeCN-H_2_O (4:1) for 40 min at a flow rate 1 mL/min. The peaks were detected with evaporative light scattering detector and D-glucose (Sigma, St. Louis., MO, USA) showed the retention time at 9.277 min. 

### 3.5. Chromatographic Conditions

The HPLC fingerprint analysis was conducted on an Agilent HPLC system with a diode array detector (DAD) and Thermo C18 column (4.6 × 250 mm, 5 μm) maintained at 30 °C. The mobile phases were acetonitrile (A) and 0.1% phosphoric acid in deionized water (B) (v/v) and a flowrate of 1.0 mL/min was utilized. The gradient elution program was as follows: 0–5 min, 10% to 17% A; 5–15 min, 17% to 17% A; 15–29 min, 17% to 57% A; 29–36 min, 57% to 80% A; 36–43 min, 80% to 80% A; An injection volume of 20 μL was used for each run. The absorption spectra of the samples were recorded in the range of 190–400 nm and the detection wavelength was set at 254 nm for the establishment of fingerprints.

### 3.6. MTT Assay

The cytotoxicity of compounds isolated from the T. patula, against human gastric cancer cell (AGS) was assessed by MTT colorimetric method. Cells in DMEM medium supplemented with 10% fetal bovine serum (FBS) were incubated in a 96-well plate in the presence of different concentrations of test compounds (2.5, 5, 10, 20, 40, 80 μmol/L) at 37 °C in a 5% CO2 incubator for 24 h. Formazan crystals resolved with DMSO and then the ODS value at 492 nm of each well was measured. 5-FU were included as a positive control. 

## 4. Conclusions

In this work, two new compounds (**1** and **2**) and 14 known compounds were isolated and identified. Compounds **1** and **2** showed moderate cytotoxicity against human gastric cancer cell lines (AGS). Furthermore, chromatographic fingerprint analysis was performed for different types of leave and stem extracts to evaluate the quality and differentiate various types of SLT. 

## Figures and Tables

**Table 1:** ^13^C-NMR spectroscopic data of compounds **1** and **2** (500 MHz for ^1^H and 125 MHz for ^13^C data, J in Hz, recorded in CD_3_OD).

Position	1		2	
	δ_C_	δ_H_	δ_C_	δ_H_
2	86.4	5.48 (1H, t, 8.5)	90.0	5.27 (1H, d, 5.0)
3	34.4	3.12 (1H, dd, 8.0, 15.5), 3.38 (1H, dd, 8.0, 15.5)	81.0	5.33 (1H, d, 5.0)
4	128.6	7.68 (1H, s)	133.1	8.18 (1H, s)
5	115.0		115.8	
6	166.6		167.7	
7	98.4	6.27 (1H, s)	98.7	6.32 (1H, s)
8	167.8		167.7	
9	120.4		121.1	
10	204.2		205.0	
11	26.4	2.54 (3H, s)	26.6	2.61 (3H, s)
12	145.8		144.8	
13	113.9	5.32 (1H, s), 5.28 (1H, s)	114.5	5.36 (1H, s), 5.41 (1H, s)
14	70.0	4.49 (1H, d, 10.0), 4.22 (1H, d,12.8)	63.6	4.16 (1H, d, 15.0), 4.23 (1H, d, 10.0)
1’	103.7	4.28 (1H, d, 4.3)	105.5	4.54 (1H, d, 5.0)
2’	75.1	3.35 (1H, m)	75.3	3.17 (1H, dd, 5.0, 10.0)
3’	78.1	3.40 (1H, m)	78.4	3.36 (1H, s)
4’	71.7	3.21 (1H, m)	71.6	3.28 (1H, br s)
5’	78.0	3.13 (1H, m)	78.1	3.36 (1H, s)
6’	62.83	3.25 (2H, m)	62.8	3.73(1H, dd, 5.0, 10.0) 4.00 (1H, dd, 2.0, 11.5)

**Table 2:** Relative peak area (RPA) of the common peaks of the *T. patula.*

Peak No.	S1	S2	S3	S4	S5	S6	S7	S8	S9	S10
4	0.425	0.416	0.940	0.425	0.243	0.468	0.594	0.130	0.712	0.468
7	0.806	0.694	1.092	0.560	0.432	0.718	0.977	0.283	0.596	0.485
11	0.824	1.674	2.878	1.901	0.707	1.756	2.523	1.001	1.740	1.166
12	0.373	1.116	1.377	0.898	0.346	0.897	1.239	0.483	0.628	0.440
patuletin	1.000	1.000	1.000	1.000	1.000	1.000	1.000	1.000	1.000	1.000
20	0.388	1.841	1.934	2.137	0.683	1.966	3.057	1.223	1.626	2.995
22	0.201	1.304	1.673	1.079	0.697	1.234	2.072	0.370	0.928	0.303
24	0.289	2.620	2.942	1.918	0.721	2.037	3.036	0.369	1.102	0.558
25	0.468	0.629	1.320	1.244	0.696	0.658	0.702	0.362	1.819	0.839

Patuletin was taken as the reference peak to calculate RPA.

**Table 3:** Evaluation results of 10 batches of *T. patula* samples.

Similarity	S1	S2	S3	S4	S5	S6	S7	S8	S9	S10	R
S1	1										
S2	0.825	1									
S3	0.887	0.952	1								
S4	0.876	0.954	0.933	1							
S5	0.894	0.907	0.892	0.926	1						
S6	0.868	0.944	0.927	0.949	0.943	1					
S7	0.849	0.963	0.933	0.976	0.898	0.952	1				
S8	0.884	0.882	0.874	0.922	0.946	0.939	0.897	1			
S9	0.921	0.879	0.952	0.911	0.906	0.897	0.878	0.895	1		
S10	0.785	0.845	0.825	0.872	0.858	0.884	0.841	0.928	0.855	1	
R	0.915	0.964	0.963	0.979	0.958	0.977	0.967	0.958	0.949	0.910	1

**Table 4:** The human gastric cancer cell inhibitory activities of isolates.

Compounds	IC_50_ (μmol/L)
**1**	41.2 (25.94–70.53)
**2** 5-Fluorouracil	30.4 (9.13–59.06) 11.8 (−0.63–20.78)

IC_50_ was afforded with confidence interval (*n* = 3); C.I., 95% confidence interval; positive control, 5-Fluorouracil.

## Appendix A

*HMBC* *spectral data of compound 1 and compound 2*

**Table A1:** HMBC spectroscopic data of compounds **1** and **2** (500 MHz for ^1^H and 125 MHz for ^13^C Data, J in Hz, recorded in CD3OD).

Position	1		2		
	δ_H_	HMBC	δ_H_	HMBC	NOESY
2	5.48 (1H, t, 8.5)	C-3,C-9,C-12,C-13,C-14,	5.17 (1H, d, 5.0)	C-12,C-13,C-14	H-3,H-14, H-1’
3	3.12 (1H, dd, 8.0, 15.5), 3.38 (1H, dd, 8.0, 15.5)	C-2,C-4,C-8,C-9,C-12	5.33 (1H, d, 5.0)	C-1’,C-8	H-3, H-4, H-13, H-14
4	7.68 (1H, s)	C-3, C-5, C-6, C-7, C-8, C-9	8.18 (1H, s)	C-8,C-10	H-11, H-1’
5					
6					
7	6.27 (1H, s)	C-5,C-6,C-8,C-9	6.32 (1H, s)	C-5,C-6,C-9	
8					
9					
10					
11	2.54 (3H, s)	C-5, C-10	2.61 (3H, s)	C-5,C10	
12					
13	5.32 (1H, s), 5.28 (1H, s)	C-2, C-12, C-14	5.36 (1H, s), 5.41 (1H, s)	C-2,C12,C-14	H-14
14	4.49 (1H, d, 10.0), 4.22 (1H, d, 12.8)	C-1’, C-2, C-12, C-13	4.16 (1H, d, 15.0), 4.23 (1H, d, 10.0)	C2,C-12,C-13	H-2, H-13, H-1’
1’	4.28 (1H, d, 4.3)	C-14, C-2’	4.54 (1H, d, 5.0)	C-3	H-3, H-4, H-14
2’	3.35 (1H, m)	C-1’, C-3’	3.17 (1H, dd, 5.0, 10.0)	C-1’, C-3’	
3’	3.40 (1H, m)	C-2’, C-4’	3.36 (1H, s)	C-2’	
4’	3.21 (1H, m)	C-3’, C-5’	3.28 (1H, br s)	C-3’, C-6’	
5’	3.13 (1H, m)	C-4’, C-5’, C-6’	3.36 (1H, s)	C-4’	
6’	3.25 (2H, m)	C-5’	3.73(1H, dd, 5.0, 10.0) 4.00 (1H, dd, 2.0, 11.5)	C-4’, C-5’	H-4’

*Method* *Validation*

The precision was measured by analyzing the five replicates of the sample solutions (Sample 1) continuously within one day. The relative standard deviation (RSD) values of relative retention time (RRT) and peak area (RPA) of the fourteen markers did not exceed 0.49% and 6.22%, respectively. The stability test was determined and the RSD values of RRT and RPA were below 0.52% and 6.25%. Moreover, five independently solutions of the same sample (Sample 1) were analyzed to verify the repeatability. The RSD values of RRT and RPA were less than 0.59% and 4.70%, respectively. The results of the precision, stability, and repeatability rates are shown in Table A2.

**Table A2:** Analytical results of precision, stability, and repeatability rates of fourteen components in the *T. patula*.

Peak No.	RSD of RPA (%)			RSD of RRT (%)		
	Precision	Stability	Repeatability	Precision	Stability	Repeatability
5	4.397	4.769	3.401	0.492	0.458	0.246
6	3.504	5.883	1.574	0.321	0.238	0.258
11	3.343	6.253	3.652	0.375	0.522	0.586
15	4.840	4.976	4.701	0.125	0.131	0.098
18(S)	0.000	0.000	0.000	0.000	0.000	0.000
25	6.224	1.351	3.806	0.064	0.049	0.052

*The pictures* *of three types of flowers from stems and leaves of T. patula*
